# The Influence of Social Structure, Habitat, and Host Traits on the Transmission of *Escherichia coli* in Wild Elephants

**DOI:** 10.1371/journal.pone.0093408

**Published:** 2014-04-04

**Authors:** Patrick I. Chiyo, Laura E. Grieneisen, George Wittemyer, Cynthia J. Moss, Phyllis C. Lee, Iain Douglas-Hamilton, Elizabeth A. Archie

**Affiliations:** 1 Department of Biological Sciences, University of Notre Dame, Notre Dame, Indiana, United States of America; 2 Department of Fish, Wildlife and Conservation Biology, Colorado State University, Fort Collins, Colorado, United States of America; 3 Amboseli Trust for Elephants, Langata, Nairobi, Kenya; 4 Behaviour and Evolution Research Group, Department of Psychology, University of Stirling, Scotland, United Kingdom; 5 Save the Elephants, Nairobi, Kenya; University of Illinois at Urbana-Champaign, United States of America

## Abstract

Social structure is proposed to influence the transmission of both directly and environmentally transmitted infectious agents. However in natural populations, many other factors also influence transmission, including variation in individual susceptibility and aspects of the environment that promote or inhibit exposure to infection. We used a population genetic approach to investigate the effects of social structure, environment, and host traits on the transmission of *Escherichia coli* infecting two populations of wild elephants: one in Amboseli National Park and another in Samburu National Reserve, Kenya. If *E. coli* transmission is strongly influenced by elephant social structure, *E. coli* infecting elephants from the same social group should be genetically more similar than *E. coli* sampled from members of different social groups. However, we found no support for this prediction. Instead, *E. coli* was panmictic across social groups, and transmission patterns were largely dominated by habitat and host traits. For instance, habitat overlap between elephant social groups predicted *E. coli* genetic similarity, but only in the relatively drier habitat of Samburu, and not in Amboseli, where the habitat contains large, permanent swamps. In terms of host traits, adult males were infected with more diverse haplotypes, and males were slightly more likely to harbor strains with higher pathogenic potential, as compared to adult females. In addition, elephants from similar birth cohorts were infected with genetically more similar *E. coli* than elephants more disparate in age. This age-structured transmission may be driven by temporal shifts in genetic structure of *E. coli* in the environment and the effects of age on bacterial colonization. Together, our results support the idea that, in elephants, social structure often will not exhibit strong effects on the transmission of generalist, fecal-oral transmitted bacteria. We discuss our results in the context of social, environmental, and host-related factors that influence transmission patterns.

## Introduction

Social structure is thought to play a profound role in the transmission of both directly and environmentally transmitted infectious agents [1], [2], [3], [4]. This is because social structure–including patterns of affiliation, mating behavior, dispersal, and territoriality–determines contact and habitat use among members of a population. There is strong evidence that social structure can influence the transmission of directly transmitted organisms [5]–[7], but social structure can also be important for environmentally transmitted agents. For instance, for fecal-oral transmitted organisms, members of the same social unit tend to use the same foraging areas or water sources; hence, hosts might be most often exposed to fecal contamination from members of their own social group [8], [9], [10], [11]. Understanding when and how social structure influences transmission is important because it has implications for population management and the evolutionary costs and benefits of social behavior [3], [4], [5], [12], [13].

To date, most support for socially structured transmission of infectious agents comes from theoretical models [3], [12], [13], [14]. While theory predicts important effects of sociality on transmission, empirical evidence remains relatively scarce (but see [9], [11], [15]). Hence, for most host species and infectious agents, it is unclear how important social structure is compared to other factors that also influence transmission patterns. Here we consider four such factors that may obscure or enhance the signature of host social structure on transmission [4], [16], [17]. First, host specificity and the transmission mode of the infectious agent should determine how strongly its transmission is influenced by the social structure of a single host species. Infectious agents that are specific to a single host species and are transmitted by physical contact among hosts are more likely to reflect host social structure than generalist agents transmitted via environments or vectors. Second, host traits such as age or sex may influence differences in susceptibility and hence transmission. For instance, immune responses can change with age; hence, some age classes may dominate transmission more than others [18], [19]. In terms of host sex, males are less immunocompetent than females in many vertebrates [20], [21], [22], [23], [24], and sex-specific differences in behavior may also lead to sex-specific patterns of transmission [21], [22], [23]. Third, competitive and facilitative ecological interactions among parasites or infectious agents may influence infection and the establishment of different parasites within hosts [25], [26]. Among humans for example, commensal gut bacteria that colonize early may establish themselves as the dominant bacterial community, which may influence the establishment of late colonizers [25], [27]. Fourth, for environmentally transmitted agents, aspects of the environment may enhance or obscure socially structured transmission. For instance, habitats that are suitable for bacterial proliferation (e.g. moist, warm places) may increase the length of time that bacteria can survive in the environment and enhance the opportunities for transmission. Hence, socially structured transmission may be dominated by interactions within a few suitable transmission hotspots in the habitat [28], [29].

In this study, we used population genetic information on *Escherichia coli* to test the influence of social structure, environment, and host traits on patterns of bacterial transmission in wild African savannah elephants, *Loxodonta africana* (Blumenbach, 1870). African elephants live in fission-fusion societies where individuals belong to predictable social groups, but the strength of associations between individuals and groups can vary depending on ecological conditions [30], [31]. The basic social unit, called a “core” or “family” group, can include up to fifty adult females and their immature offspring [31], [32], [33]. At maturity, males disperse from their natal family and range widely across the population, never permanently joining another family group [34], [35]. Over the course of hours, days, or weeks, families may fission into subgroups, or they might fuse together with members of other families. Members of the same family typically spend 60% to 90% of their time together in the same group, while members of different families typically spend much less time together, ranging from zero to 40% [30], [32]. Elephants are not territorial, but families occupy predictable home ranges that overlap with a fraction of the other families in the population [36]. Thus, there are several behaviorally mediated traits that potentially influence transmission in this species.

*E. coli* is an enteric commensal and an occasional pathogen in many vertebrates. It is transmitted between hosts through both direct physical contact and the ingestion of fecal-contaminated food and water [37], [38]. *E. coli* is a common model organism to study patterns of bacterial transmission in wildlife because it is easy to sample individual *E. coli* isolates from fecal samples [10], [39], [40]. Furthermore, these isolates can be genotyped using multi-locus markers developed for strain typing and population genetics [41]. In addition, *E. coli* reproduce clonally, and recombination–i.e., the exchange of genes or gene segments with other bacteria–is usually too low to obscure its clonal structure and transmission patterns [42], [43]. With respect elephant management, disease dynamics in elephants is understudied and understanding the transmission of commensal *E. coli* may lend insight into the spread of other, more harmful fecal-oral transmitted microbes, such as *Salmonella* sp. [44], [45].

Prior studies have found that host social structure can be correlated with *E. coli* transmission patterns [9], [10], [46], [47]. For instance in giraffe, hosts that were more closely linked in a social network were more likely to be infected with similar sub-types of *E. coli* than more distantly connected hosts [9]. In humans, members of the same household are more likely to be infected with similar stains of *E. coli* than members of different households [47]. Similarly in wild gorillas, members of the same social group harbor more similar *E. coli* than members of different groups [10]. However, several variables may obscure the signal of socially structured transmission [48]. For instance, *E. coli* infects many vertebrate species; hence, its population structure may often not be heavily influenced by the social structure of a single host species [49], [50]. In addition, *E. coli* can persist for several days to several years in water, sediment, and soil [51], [52], and habitats that are suitable for bacterial proliferation may increase opportunities for recombination [53] and transmission. Furthermore, *E. coli* is a member of the gut microbiome, and host age and ecological interactions among bacteria may influence which isolates establish in a given host [54], [55].

Our main objective was to test whether host social structure plays a detectable role in shaping patterns of *E. coli* transmission among wild elephants. We examined the role of social structure in the context of several other factors that may also influence *E. coli* transmission, including aspects of the habitat, host sex, and host age. We inferred patterns of transmission using the population genetic structure of *E. coli* isolated from elephants living in two populations: the Amboseli ecosystem in southern Kenya, and the Samburu-Laikipia ecosystem in central northern Kenya. Although both populations occupy areas with a similar climate, they differ in that the Amboseli population has a permanent swamp in their core habitat, while the Samburu population does not. We addressed two main questions: 1) Do host social structure and patterns of habitat use predict the population genetic structure of *E. coli*? And 2) what other aspects of individual hosts or their environments are important in shaping the population genetic structure of *E. coli*? If social structure influences *E. coli* transmission, we predicted that: a) *E. coli* infecting elephants from the same family should be genetically more similar than *E. coli* infecting members of different families, and b) the degree of range overlap would predict the degree of *E. coli* genetic similarity between families. In terms of host traits, we tested whether male elephants, which have larger ranges than females and possible differences in immune function, harbored genetically more diverse or potentially more pathogenic *E. coli* than female elephants. Finally, we explored age-related effects on patterns of *E. coli* infection, and specifically tested the prediction that elephants more similar in age are infected with genetically more similar *E. coli*, compared to elephants more different in age.

## Methods and Materials

### Ethics Statement

All protocols were noninvasive and adhered to the guidelines approved by the Institutional Animal Care & Use Committee (IACUC) of the University of Notre Dame. In Kenya, permission to conduct research was granted by the Kenyan government through research permit number NCST RRI/12/1/MAS/118/4.

### Study Area and Host Populations

Study subjects were wild elephants living in the Amboseli ecosystem (8,000 km^2^), in southern Kenya, and the Samburu-Laikipia ecosystem (37,360 km^2^), which lies about 390 km to the north of Amboseli, in central northern Kenya. Elephant migration between these parks is probably currently rare, given the level of urban development between these locations. Both populations have been subject of long-term research; elephants in Amboseli have been monitored continuously for over 40 years, while elephants in Samburu have been monitored continuously for over 15 years [56], [57]. In both populations, individuals can be reliably identified, and for most animals born since the onset of monitoring, age is known ±2 weeks. For animals older than the onset of monitoring (wild elephants can live 65 years or more), age is estimated using well-developed morphological metrics, including shoulder height, hind footprint lengths and body shape [58], [59]. Ages were considered accurate within a few months to a few years, depending on the age class of the animal [60], [61].

There are several environmental similarities between the two habitats. In both Amboseli and Samburu, the elephants use the protected areas as core areas while making regular forays outside these protected areas to forage [62], [63], [64]. The core range of the Amboseli elephant population (∼1400 elephants) is Amboseli National Park, which covers 390 km^2^ between 1° 37′–3°13′ S and 35° 49′–38° 00′ E [56]. The core range of the Samburu elephant population (∼900 elephants) is the Samburu and Buffalo Springs National Reserves, which covers 220 km^2^ between 00° 30′–00° 80′ N, and 37°–38°E [65]. Both habitats are classified as semi-arid savannah with mixed open and bushy grassland and sparse woodland. Both habitats also contain other species of vertebrate hosts that are likely to be infected with *E. coli* and that may influence transmission patterns. Both habitats have similar patterns of seasonality and rainfall; rainfall occurs between November and May, and average annual rainfall is 340 mm in Amboseli and 360 mm in Samburu [66], [67].

While there are several ecological similarities between Amboseli and Samburu, there is one difference that may have implications for *E. coli* transmission: Amboseli has perennial swamps that cover about 12% of the National Park, whereas the Samburu has a free-flowing river. The presence of extensive, permanent, water-fed swamps in Amboseli may provide a conducive environment for the proliferation and transmission of *E.coli* [53], [68].

### Defining Family Groups and Measuring Range Overlap between Groups in Protected Areas

To understand how social structure influenced the transmission of *E. coli*, we measured the genetic structure of *E. coli* isolates sampled from elephants living in 10 families in Amboseli and 5 families in Samburu (Table S1). Families were defined as a collection of adult females and their juvenile offspring that exhibited consistent associations, coordinated activities, and high rates of affiliative behaviors exchanged exclusively among family members [32], [33], [69]. In Amboseli, the families in our study contained between 7 and 31 adult females and their juvenile offspring, with an average size of ∼21 individuals. In Samburu, families ranged in size from 10 to 21 animals, with an average size of ∼14 individuals.

To test whether host occupancy patterns influenced the transmission of *E. coli* between family groups, the range (km^2^) of each group within the protected areas and the percent of spatial overlap between ranges in the protected areas were estimated using GPS locations of sightings of family members collected over a five-year period prior to *E. coli* sampling (March 2005 to March 2010 in Amboseli and January 2006 to December 2011 in Samburu). We chose a five-year span to maximize the number of sightings for each family (Table S1). Elephant GPS locations were collected while driving on roads within either Amboseli National Park or the Samburu and Buffalo Springs National Reserves. When an elephant group was sighted, we left the road and approached the group to record a GPS point representing the group’s location. These range estimates under-represent the total area used by the elephants because they lack data on the elephants’ ranges outside the protected areas. However, in both Amboseli and Samburu, the protected areas contain reliable food and water and are relatively safe from human threats; hence they comprise a major proportion of each family’s range, especially in the dry season [62], [63], [64]. Moreover, in both populations, the elephants often follow a diurnal pattern whereby they range within the park during the day and radiate beyond the park’s borders at night, with lower range overlap between families outside than inside the park [62], [63], [64]. As a result, the elephant ranging patterns we measured represent the areas of highest elephant density, greatest spatial overlap between elephant families, and perhaps the highest potential for bacterial transmission [62], [64]; however we cannot exclude the possibility that ranging patterns outside the protected areas also influenced the transmission patterns we observed.

**Figure 1:**
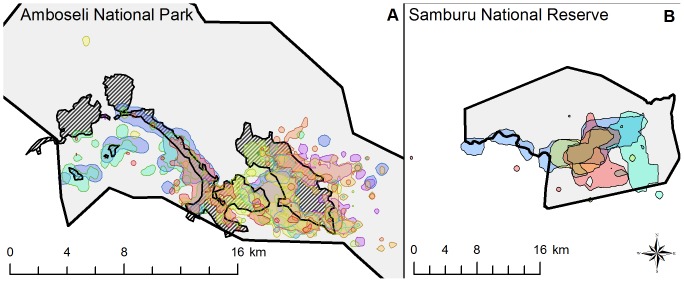
Ranges of elephant families within (A) Amboseli NP and (B) Samburu NR. The outline of each protected area is depicted by a thick black line, and the protected area is shaded in light grey. The ranging patterns for each family are shown by different colors, and areas of overlap are shown as a blend of colors of the different overlapping family groups. Swamps in Amboseli are represented by a black striped pattern.

From these GPS data, we used a kernel density estimator in BIOTAS software (Version 2.0a.3.8) to calculate a “protected area range” for each family from 95% of GPS locations by eliminating 5% of the locations that were spatial outliers (Figure 1). To minimize the influence of large differences in range size between families, we calculated percent overlap in protected areas as the ratio of area shared by two families divided by the geometric mean of the two ranges. Compared to the arithmetic mean, the geometric mean minimizes bias in percent overlap caused by large differences in a pair of ranges [70], [71]. In Amboseli, we also estimated the area (km^2^) of each family’s range in swamp habitat, and the percent overlap among pairs of families in swamp habitat (Samburu lacked swamp habitat).

### Escherichia coli Collection, DNA Extraction, and Genotyping

*E. coli* was cultured from fecal samples collected from known elephants during intensive 1-month sampling efforts during July 2010 in Amboseli and June 2011 in Samburu (Table S1). When members of a study group were located, we approached them in a vehicle and waited to collect fecal samples. As soon as a sample was produced by a known individual, we drove close to the sample, donned sterile gloves, and used sterile tongue depressors to collect pieces from the outside of the fecal bolus into sterile tubes (we sampled from the outside of the bolus to maximize the region in contact with the intestinal wall, where we suspected *E. coli* might be most likely to be found). Samples were typically collected within 10 minutes of defecation, and we avoided all sections of the fecal sample that may have made contact with the ground. Samples were kept at ambient temperature for 1 to 4 hours until we cultured and collected individual *E. coli* isolates from each sample. Specifically, we lit a candle in the workspace to create an up-draft. We then lightly touched the surface of the fecal sample with a flame-sterilized metal loop to collect a small amount of feces (an amount barely visible to the naked eye), and streaked the loop onto individual MacConkey agar plates (MacConkey agar is selective for gram negative bacteria and indicates the presence of lactose fermentation). Samples were incubated at 37°C for 12–24 h to allow colonies to grow. We collected one fecal sample per individual, and for each fecal sample, we used sterile toothpicks to collect 4 to 10 putative *E. coli* colonies (i.e. colonies matching the expected colony phenotype for *E. coli* on MacConkey agar: round, uniform, bright pink colonies). After collection, each putative *E. coli* colony was transferred to its own tube, containing 95% ethanol. We refer to these preserved colonies as “isolates”. Each putative *E. coli* isolate was stored at room temperature for up to four weeks prior to genetic analysis.

We genotyped each putative *E. coli* isolate using seven multi-locus sequence typing (MLST) loci [41] consisting of the following genes: *adk* (adenylate kinase), *fumC* (fumarate hydratase), *gyrB* (DNA gyrase) *icd* (isocitrate dehydrogenase), *mdh* (malate dehydrogenase), *uidA* (beta-glucuronidase) and *ClpX* (caseinolytic protease × homolog) [72], [73]. The combined length of these sequences was ∼3700 base pairs. We choose these genes because they have sufficient variation for discriminating bacterial isolates and have been used to track and monitor population genetics and epidemiology of a number of bacterial pathogens, including pathogenic *E. coli*. We extracted DNA from 2 to 4 individual *E. coli* isolates per Amboseli elephant and 4 *E. coli* isolates per Samburu elephant. DNA was extracted using DNAeasy (Qiagen tissue kit) following the manufacturer’s instructions. We amplified each MLST marker using the polymerase chain reaction (PCR) conditions described in [72], [73]. Positive PCR reactions were sequenced in the reverse and forward directions using Dye Terminator Cycle Sequencing (Applied Biosystems). Sequences were inspected and cleaned using Sequencher software (version 4.9). A small fraction of sequences with multiple ambiguous nucleotides were discarded from further analyses. Clean sequences were subjected to NCBI BLAST to confirm that the isolates were *E. coli* (>95% of isolates were assigned to *E. coli*; others were excluded from further analyses).

### Population Genetic and Statistical Analyses

We concatenated the sequences from each MLST gene for each *E. coli* isolate. An *E. coli* isolate with a distinct concatenated sequence is henceforth referred to as a “haplotype”. Prior research has shown that *E. coli* haplotypes can belong to one of four major phylogenetic groups, referred to as A, B1, B2, and D [74]. Phylogroups A, B2, and D, are common in humans, while phylogroup B1 is common in animals and abiotic environments [43], [68], [75]. Members of phylogroups B2 and D are most often commensal, but are more likely to be carriers of extra-intestinal virulence factors than are phylogroups A or B1 [76], [77]. We assigned our haplotypes to phylogroups by comparing our haplotypes with an *E. coli* Reference Collection (ECOR) consisting of 72 sequences with known phylogroup membership [78]. We did this by constructing a neighbor-joining tree including our isolates and the 72 ECOR haplotypes in MEGA software (MEGA5.2.1).

We examined basic patterns of genetic diversity, demographic history, and recombination rates since these forces might influence the population structure of *E. coli* and hence our ability to detect transmission patterns. We calculated nucleotide diversities and Tajima’s D in Arlequin (Version 3.5.1.3) [79]. We estimated mutation rates, recombination rates, and population history using Bayesian inference implemented in ClonalFrame (Version 1.1) [80]. We calculated two measures of the recombination rate: ρ/θ, which measures the relative frequency of occurrence of recombination and mutation in the population, and R/M, which measures the relative contribution of recombination and mutation to nucleotide substitution. Four independent runs of ClonalFrame were performed each consisting of 400,000 MCMC iterations, with the first half discarded as burn-in. Convergence and mixing of the MCMC for each simulation run was found to be satisfactory after visual inspection and after conducting the Gelman-Rubins statistics for all model parameters for convergence between any pair of runs.

To test the influence of host social structure on *E. coli* population genetic structure, we used analysis of molecular variance (AMOVA) in Arlequin to partition genetic variance in *E. coli* sampled from adult female and juvenile elephants within and between host populations, family groups and individual hosts. Measuring the population genetic structure of bacteria can be challenging because the clonal structure may vary depending on the rate of recombination. Hence, we performed AMOVA and calculated F_ST_ using two different measures of genetic distance, each with their own assumptions. First, to account for evolutionary distance between haplotypes caused by mutations and recombination events, we calculated genetic distance based on the ClonalFrame analysis. Specifically, we measured “patristic distance” or the total branch lengths between each pair of isolates calculated from a 50% consensus evolutionary tree, assuming uniform nucleotide substitution and recombination using a Bayesian analysis in ClonalFrame. This approach to inferring transmission assumes that genetically more similar haplotypes share more recent common ancestors and more recent transmission events. Second, we measured “haplotype distance” by performing AMOVA using classic haplotype frequency-based measures of genetic variance, which ignore phylogenetic distance between haplotypes. This measure of genetic distance, which is based on the proportion of shared haplotypes, may be better at capturing patterns of transmission when evolutionary rates are high.

We next tested whether range overlap in protected areas influenced *E. coli* genetic similarity between family groups. As in the AMOVA’s above, *E. coli* isolates sampled from adult males were excluded from these analyses. Mantel tests were used to assess the correlation between matrices of the percent of range overlap and matrices of genetic differentiation (F_ST_) based on patristic distance and haplotype distance between *E. coli* sampled from different family groups. In Amboseli we also conducted a second analysis using percent range overlap in swamps alone.

We next performed three analyses to understand the associations between host traits (sex and age) and *E. coli* genetic and phylogroup structure. First, using samples from Samburu only, we tested whether independent adult males (N = 7; males who were no longer regularly associated with a single family) were infected with more diverse *E. coli* than adult females. Specifically, we performed a regression analysis with host sex and age as predictor variables and average nucleotide diversity among *E. coli* isolates from within a host as the dependent variable. When a covariance analysis showed that age was not an important predictor of *E. coli* nucleotide diversity within individuals, but sex was, we carried out an independent sample t-test between genetic distances of *E. coli* isolates from adult male and adult female hosts.

Second, we used generalized linear mixed modeling (GLMM) to test whether host age or sex influenced the tendency for individuals to be infected with each phylogroup. We expected males to be more often infected with phylogroups associated with greater pathogenic potential (phylogroups B2 and D). We constructed a Poisson GLMM for each *E. coli* phylogroup. To test whether the proportion of isolates belonging to a given phylogroup changed as a function of host age and sex, we used the number of *E. coli* isolates belonging to a phylogroup from each individual elephant as the response variable and the total number of *E. coli* isolates obtained from that individual host as an offset variable. The use of the offset variable allowed us to model counts of each phylogroup as proportions. We used host sex and age as fixed factors, and host population as a random factor. The effect of age and sex on the proportion of isolates for some phylogroups did not vary by host population (e.g. A and B1). For these phylogroups, we instead ran a generalized linear model (GLM) that retained all the fixed factors but without including population as a random factor.

Third, we tested the prediction that same-aged elephants are infected with genetically more similar strains of *E. coli*, as compared to animals more disparate in age. Specifically, we used Mantel tests to correlate pairwise matrices of difference in age with matrices of genetic distance between *E. coli* isolates sampled from that pair of elephants.

## Results

### Basic Patterns of Genetic Diversity, Phylogroup Membership, and Sequence Evolution

We assessed the population genetic structure of *E. coli* using 210 *E. coli* isolates from 85 adult female and juvenile Amboseli elephants, and 143 *E. coli* isolates from 36 Samburu elephants (7 males and 29 adult females and juveniles; Table S1; NCBI GenBank Accession numbers KJ078651- KJ081101). We observed 140 total haplotypes; 41 of these haplotypes (29%) occurred in multiple hosts. In Amboseli, we found 93 distinct haplotypes in 85 elephants; in Samburu we found 60 haplotypes in 36 elephants. Two-thirds of *E. coli* isolates were assigned to phylogroup B1, while the remaining third was divided among phylogroups A, B2, D, and a small unassigned class (Table S2). Nucleotide diversities were similar in Amboseli and Samburu, with an average percent of nucleotide differentiation around 1.2% (Table 1). ClonalFrame analyses indicated that recombination was the dominant evolutionary force in *E. coli*, and the relative contribution of recombination was about 3 times the contribution of mutation (Table S3). In support for the idea that aquatic environments promote recombination, the relative contribution of recombination versus mutation was about 1.5 times higher in Amboseli as compared to Samburu (Table S3).

**Table 1:** Basic genetic diversity statistics for *E. coli* sampled from elephants in Amboseli and Samburu.

Parameters	Amboseli	Samburu	Combined
Number of isolates	210	143	353
Number of segregating sites	277	330	388
Number of haplotypes	93	60	140
Mean population Nucleotide diversity ± SD	0.012±0.006	0.013±0.006	0.012±0.006
Mean Nucleotide diversity within individuals ± SD	0.009±0.007	0.007±0.005	0.008±0.007
Tajima’s D (p-value)	−0.574 (0.331)	−0.686 (0.287)	−0.795 (0.228)

### Neither Host Population nor Social Group were Significant Barriers to E. coli Transmission

Before exploring the effects of elephant social structure on *E. coli* genetic structure, we first investigated how genetic variation in *E. coli* was partitioned within and between host populations and individual hosts (Table 2). We performed AMOVA on two measures of genetic distance: “patristic distance” from ClonalFrame, which estimates phylogenetic distance between haplotypes controlling for recombination, and “haplotype distance”, which ignores phylogenetic distances between haplotypes (see methods). As expected for *E. coli* [49], [50], we found little genetic differentiation in *E. coli* sampled from these two populations, despite being separated by nearly 400 km (Table 2). For haplotype distance, around 1% of genetic variance was explained by host population, indicating that there are small but significant differences in the frequencies of some *E. coli* haplotypes in Amboseli versus Samburu (Table 2). For instance, families of elephants from the same population (i.e. either Amboseli or Samburu) tended to share a greater proportion of haplotypes compared to families from different populations (average proportion of haplotypes shared between families in the same population ± SE = 7.6% ±0.6%; average proportion of haplotypes shared between families in different populations ± SE = 3.8% ±0.6%). However, the great majority of genetic variance in *E. coli* (99%) was found within and between individual elephants living in the same population. Indeed, individual hosts tended to contain diverse haplotypes; for instance, within-host nucleotide diversities were around 1%, and the average number of pairwise nucleotide differences between isolates sampled from the same host was 30.8±25.1 in Amboseli and 24.4±18.8 in Samburu (Table 1).

**Table 2:** Results of an AMOVA depicting the contribution of host populations and host individuals to the partitioning of genetic variation in *E. coli* isolates.

Source of variation	d.f.	Sum of squares	Variance components	Percentage variation	F_ST_	P
***Patristic distance***						
Among host populations	1	0.753	0.002	0.68	0.007	0.094
Among individual hosts within host populations	119	49.492	0.095	40.46	0.407	0.000
Within individual hosts	232	32.181	0.139	58.86	0.411	0.000
***Haplotype distance***						
Among host populations	1	1.608	0.004	0.89	0.009	0.000
Among individual hosts within host populations	119	90.265	0.138	27.57	0.278	0.000
Within individual hosts	232	82.983	0.358	71.54	0.285	0.000

We next tested whether families represented significant barriers to *E. coli* gene flow. If elephants are more likely to be infected with *E. coli* from members of their own family, as compared to members of other families, then *E. coli* sampled from members of the same family should be genetically more similar than *E. coli* sampled from members of different families. However, we found no support for this prediction. Instead, family groups did not explain a significant fraction of the genetic variance in *E. coli* in either Amboseli or Samburu, regardless of the measure of genetic distance (Tables 3 and 4; P = 0.688 in Amboseli; P = 0.142 in Samburu). In support, individuals were just as likely to share haplotypes with members of different families as they were with members of their own family (average proportion of haplotypes shared between members of the same family ± SE = 8.3% ±1.4%; average proportion of haplotypes shared between members of different families, but from the same population ± SE = 7.6% ±0.7%).

**Table 3:** Results of an AMOVA depicting the contribution of elephant social groups (adult females and juveniles only) to the partitioning of genetic variation of the *E. coli* isolates from Amboseli elephants.

Source of variation	d.f.	Sum of squares	Variance components	Percentage of variation	F_ST_	P
***Patristic distance***						
Among family groups	9	4.374	−0.001	−0.40	−0.004	0.688
Among individual hosts within family groups	75	35.457	0.111	35.51	0.354	<0.001
Within individual hosts	125	25.383	0.203	64.89	0.351	<0.001
***Haplotype distance***						
Among family groups	9	5.975	0.000	−0.09	−0.001	0.605
Among individual hosts within family groups	75	47.844	0.099	19.99	0.200	<0.001
Within individual hosts	125	49.667	0.397	80.1	0.199	<0.001

**Table 4:** Results of an AMOVA depicting the contribution of elephant social groups (adult females and juveniles only) to the partitioning of genetic variation of *E. coli* isolates from Samburu elephants.

Source of variation	d.f.	Sum of squares	Variance components	Percentage of variation	F_ST_	P
***Patristic distance***						
Among family groups	4	4.251	0.009	2.57	0.026	0.142
Among individual hosts within family groups	24	20.565	0.175	51.11	0.525	0.000
Within individual hosts	87	13.781	0.158	46.33	0.537	0.000
***Haplotype distance***						
Among family groups	4	4.782	0.006	1.12	0.011	0.116
Among individual hosts within family groups	24	25.539	0.192	38.84	0.393	0.000
Within individual hosts	87	25.817	0.297	60.04	0.400	0.000

### Range Overlap between Family Groups in Protected Areas is a Mixed Predictor of *E. coli* Gene Flow

To test whether range overlap in protected areas was correlated with *E. coli* genetic similarity, we first examined the degree of range overlap between elephant families within the two reserves. Within protected areas, family ranges were larger and had higher percent overlap with each other in Samburu compared to Amboseli (Figure 1; Samburu: average ± SD size = 53.1±16.3 km^2^; average percent ± SD overlap = 50.2% ±10.1%; Amboseli: average ± SD size = 20.7±7.0 km^2^; average percent ± SD overlap = 22.6% ±18.7%). In Amboseli, elephant families had considerable overlap with swamp, which constituted, on average 70% of a family’s range within this protected area (Figure 1; Average area in swamp ± SD = 2.425±2.253 km^2^; range = 0.051 to 8.240 km^2^). In terms of range overlap between families, the mean ± SD percent overlap in swamp was 27.86% ±22.87% (range: 0.95% to 80.84%).

**Figure 2:**
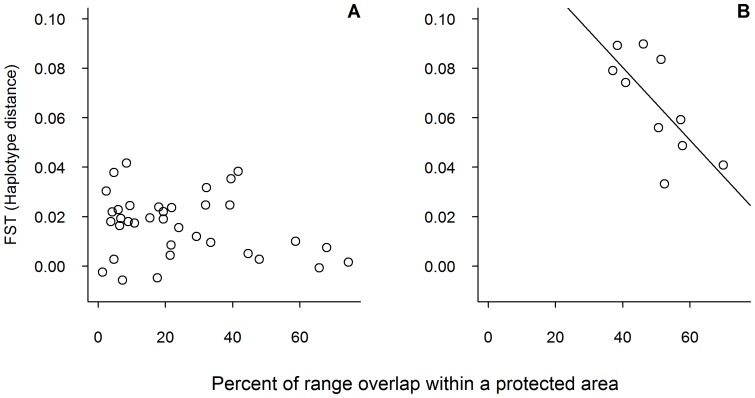
*E. coli* genetic distance as a function of range overlap between elephant families. (A) Depicts data from Amboseli and (B) depicts data from Samburu. The relationship between percent overlap and F_ST_ was statistically significant in Samburu, but not Amboseli. Plots are for visualization purposes only; statistical analyses were performed using Mantel tests (see text for details). Analyses include data from adult female and juvenile elephants only.

We expected that higher range overlap in protected areas would be associated with greater *E. coli* genetic similarity between families. We found mixed support for this prediction. In Amboseli, the percent of range overlap was not correlated with *E. coli* genetic similarity, whether we tested this relationship for complete ranges (Figure 2a; patristic F_ST_: r_s_ = 0.085, P = 0.612; haplotype F_ST_: r_s_ = −0.148, P = 0.387), or only for range overlap in swamp (patristic F_ST_: r_s_ = −0.048, P = 0.777; haplotype F_ST_, r_s_ = −0.264, P = 0.116). However, we did observe a significant relationship between range overlap between families and genetic similarity of *E. coli* in Samburu (Figure 2b; patristic F_ST_: r_s_ = −0.770, P = 0.013; and haplotype F_ST_: r_s_ = −0.697, P = 0.031).

### Adult Male Elephants are Infected with Genetically More Diverse and Potentially More Pathogenic E. coli than Adult Females

We predicted that adult male elephants might harbor greater genetic diversity of *E. coli* than adult females. In support, we found that adult males were infected with genetically more diverse strains than females as measured by nucleotide diversity (mean nucleotide diversity for females = 0.006±0.005, for males = 0.011±0.005; t = −2.743, df = 25 P = 0.011, Figure S1). We also predicted that males might be more likely to be infected with phylogroups B2 and D, which are more likely to carry virulence factors than other phylogroups. In support, we found that adult males harbored a significantly higher proportion of phylogroup D than adult females (Table 5). We also observed a non-significant trend for males to be infected with a higher proportion of phylogroup B2 (Table 5).

**Table 5:** Results of generalized linear models (model 1 & 2) and generalized linear mixed effects models (models 3, 4 & 5) showing the influence of host age and sex on the proportion of each phylogenetic group of *E. coli* in elephants.

Model	Phylogroup	Covariate	Estimate	Standard error	z value	P
Model 1	A					
		Age	−0.025	0.016	−1.530	0.126
		Sex	−0.005	0.405	−0.012	0.990
Model 2	B1					
		Age	−0.003	0.005	−0.607	0.544
		Sex	−0.225	0.164	−1.374	0.169
Model 3	B2					
		Age	0.027	0.019	1.428	0.153
		Sex	0.926	0.554	1.671	**0.095**
Model 4	D					
		Age	−0.036	0.023	−1.598	0.110
		Sex	1.069	0.451	2.368	**0.018**
Model 5	unclassified					
		Age	0.035	0.012	2.813	**0.005**
		Sex	−0.251	0.514	−0.487	0.626

### Elephants from Similar Age Cohorts were Infected with Genetically More Similar E. coli

**Figure 3:**
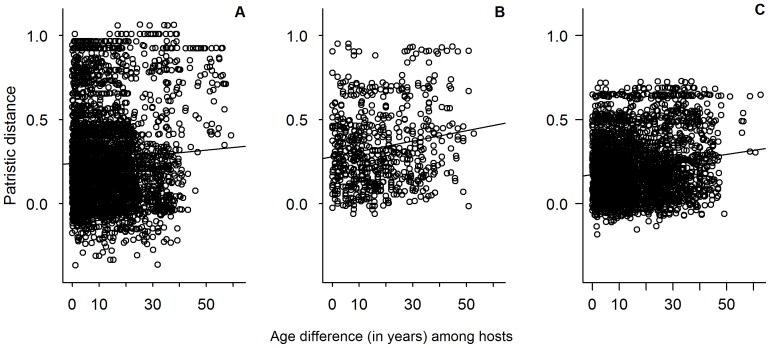
*E. coli* genetic distance as a function of age-difference among hosts. (A) Depicts the relationship between age similarity and *E. coli* genetic similarity as measured by patristic distance for pairs of elephants from Amboseli; (B) depicts these relationships for pairs of elephants from Samburu; (C) depicts these relationships for pairs of elephants where one member was drawn from each population. Plots are for visualization purposes only; statistical analyses were performed using Mantel tests (see text for details).

For many pathogens, host age plays an important role in transmission, and there is some evidence that age can influence patterns of *E. coli* infection in humans [55], [81], [82], [83]. We tested for age effects on patterns of *E. coli* infection by correlating a matrix of pairwise difference in age between pairs of elephants with a matrix of pairwise genetic distance between *E. coli* isolates infecting those elephants. We found some support for genetic structuring in *E. coli* populations based on host age. In both Amboseli and Samburu, difference in age explained a small but significant fraction of the genetic variance in *E. coli* (Figure 3; patristic distance in Amboseli: r = 0.062, P = 0.0003; patristic distance in Samburu: r = 0.170, P<0.0001; haplotype distance in Amboseli: r = 0.040, P = 0.019; haplotype distance in Samburu: r = 0.186, P<0.0001). Specifically, elephants closer in age were more likely to be infected with genetically more similar *E. coli* than elephants more different in age (Figure 3).

We also examined the correlation between *E. coli* genetic distance and host age for *E. coli* isolated from pairs of elephants from the two different populations. Interestingly, the effect of age similarity cut across elephant populations (Figure 3c). Specifically, using only pairs of elephants from the two different populations, age similarity still predicted genetic similarity of *E. coli* (patristic distance: r = 0.160, P<0.0001; haplotype distance: r = 0.124, P<0.0001). One explanation for these results is that age might predict a host’s probability of infection with a given phlyogroup, perhaps through age-specific patterns of susceptibility [55], [81], [82], [83]. However, we found only limited support for this idea; age did not predicted the probability that elephants were infected with a given phylogroup, and phylogroups were evenly distributed across hosts of different ages, but we observed that the proportion of strains that did not cluster with a specific phylogroup increased with age across both elephant populations (Table 5; Figure S2).

## Discussion

Social organization and behavior can influence the transmission of both directly [5]–[7] and environmentally transmitted infectious agents [1], [2], [4], [8], [9], [10], [11], [84]. However in natural populations, several other factors may also influence transmission, including aspects of the abiotic environment that promote or impede transmission, and host traits such as sex or age that influence individual exposure and the probability of infection [19], [22]. In this study, we used population genetic tools to explore the effects of social structure, environment, and host traits on the transmission of *E. coli* infecting wild elephants. We found little evidence for socially structured transmission; instead, the population genetic structure of *E. coli* was heavily influenced by processes occurring in the environment and at the level of individual hosts. Below we discuss our results, starting with the effects of habitat on patterns of *E. coli* transmission.

### The Influence of Social-structure and Habitat on Transmission

Many researchers have hypothesized that exposure to infectious disease is an important evolutionary cost of group living [1], [85], [86]. This hypothesis assumes that directly and environmentally transmitted agents are more likely to spread among members of the same social group than between members of different groups. While there is considerable evidence for this assumption, even for environmentally transmitted infectious agents [8], [9], [10], [11], [84], in our study we found no evidence that elephants were more likely to be infected with *E. coli* from members of their own family group than members of other families. Instead, *E. coli* isolates sampled from members of the same family were not genetically more similar compared to *E. coli* from different families, and families did not represent major barriers to *E. coli* gene flow.

These results differ somewhat from prior studies on other social species, including giraffes, baboons, gorillas, and humans, which found that individuals with more frequent social contact were more likely to be infected with more similar strains of *E. coli* [9], [10], [46], [47], [87], [88], [89]. At least four factors may explain why our results differ from some prior studies. First, variation in social organization and behavior may explain why socially structured patterns of *E. coli* transmission are more common in some species than others. During rainy periods, elephants aggregate in large groups and have physical contact between members of different families, especially during play or mating, which may promote between-group transmission. In addition, elephants have high range overlap around water sources, which may serve as environmental reservoirs for *E. coli* and promote between-group transmission. By contrast, aspects of human behavior may make *E. coli* transmission within households much more common than between households, such as high rates of physical contact between family members, hand washing, cooking and eating at home, and the use of toilets and latrines.

Second, differences in study design may have led to higher rates of within- versus between-group contact rates in prior studies compared to our study. For instance, in the studies on baboons and gorillas, researchers chose social groups with non-overlapping home ranges, which should reduce the probability that individuals would encounter fecal contamination from non-group members [10], [89]. Third, different *E. coli* phylogroups vary in their prevalence across host species and abiotic environments, and the likelihood of socially structured transmission may depend on phylogroup identity. For instance, nearly 70% of isolates from the elephants in our study were attributed to phylogroup B1, a commensal form that is known to be environmentally ubiquitous, to infect a wide variety of animals, and to have high turnover within hosts [75], [90], [91]. These traits may make it less likely to detect the effects of host social structure on transmission. In contrast, most human studies focus on pathogenic or virulent strains. Such strains are more likely to be epidemic and clonal in nature [92], [93], [94], making it easier to detect patterns of socially structured transmission.

Fourth, differences in genotyping methods may also explain why our results differed from some prior studies. In particular, two prior studies that found effects of social structure on *E. coli* transmission used the BOX-PCR method of Cesaris et al. [95] to define genetically similar isolates of *E. coli* in giraffes. Studies on *E. coli* infecting gorillas and humans used a similar approach called repetitive-element PCR [10], [96]. These approaches rely on gel- or capillary-based banding patterns, and there are fewer statistical tools to analyze such data, compared to DNA sequences of MLST markers. However, BOX-PCR and repetitive-element PCR may provide genetic information across a greater proportion of the *E. coli* genome, which may lend more power to distinguish relationships among strains. Future studies may find it valuable to compare results from these two methods of *E. coli* genotyping.

While our results indicate that elephants were not more likely to be infected by *E. coli* from members of their own family than from members of other families, we observed some support for socially structured transmission via habitat overlap [10], [40], [97]. However, this evidence was equivocal because we only observed significant patterns in one of our study populations (i.e. Samburu). One possible explanation for this mixed result is that elephant families may have substantially different patterns of habitat overlap inside versus outside protected areas; hence our measure of habitat overlap may not have accurately captured patterns of environmental exposure. However, at the population level elephant families exhibit the highest level of habitat overlap within protected areas, so we think this explanation is unlikely [63].

Another possible explanation for these mixed results is that differences in habitat moisture and the degree of habitat overlap between elephant family groups favored socially structured transmission via range overlap in Samburu, but not in Amboseli. Specifically, in Samburu, the habitat was drier, and the elephant groups exhibited a greater degree of range overlap than in Amboseli. These two forces may act in concert to increase the strength of socially structured transmission; harsh habitat may reduce *E. coli* persistence times [52], [98], while higher range overlap will increase contact between groups, ultimately leading to relatively high rates of *E. coli* transmission between elephant groups.

One final observation on socially structured transmission via range overlap: while we observed support for this hypothesis in Samburu, it is as yet unclear whether the patterns of transmission we observed between elephant groups were driven by socially mediated factors (i.e., contact with fecal material from members of different elephant families) or were instead driven by the fact that these elephant groups used the same areas and so were exposed to the same environmental sources of *E. coli* (i.e., shared habitat with alternative host species that also transmit *E. coli*). Hence, despite possible support for socially structured transmission, these results are far from conclusive about the role of social behavior in the transmission of *E. coli* between elephant groups.

### The Influence of Host Sex and Age on the Structure of *E. coli* Populations within Hosts

Most of the genetic variation in *E. coli* populations was structured within and between individual hosts, not family groups or host populations. This population structure–i.e., high gene flow between geographically distinct populations, but strong genetic differentiation between hosts–is typical of *E. coli*. For instance, researchers often find high levels of *E. coli* gene flow between host populations separated by large geographic distances [50]. Moreover, many studies have found that the majority of genetic variation in *E. coli* is structured within and between individual hosts [40], [89], [99].

For the elephants in Amboseli and Samburu, most individuals were infected with multiple, genetically diverse haplotypes, which often differed in identity and frequency from the haplotypes found infecting other members of their family or population. These patterns suggest that processes occurring at the level of individual hosts, as opposed to families or host populations, are most important in influencing the structure of *E. coli* populations. In particular, *E. coli* is a normal member of the gut microbiome, and the process of microbiome assembly–including bacterial colonization, interactions among bacterial species, and interactions with the host’s genome and immune system–may influence which *E. coli* haplotypes occur in a given host [25], [27], [100]. We identified two specific host traits that were associated with the structure of *E. coli* populations in individual hosts: host sex and age. With respect to sex, we found that adult males were infected with more genetically diverse *E. coli* and were more likely to be infected with strains from phylogroup D than were adult females. This result is based on a relatively small sample size, and it should be interpreted with caution. Moreover, phylogroup proportions are not independent of each other; however, we observed no other significant relationships between sex and phylogroup proportions, suggesting that sex is the primary predictor of phylogroup D. If true, one explanation for this result is that adult male elephants range more widely than adult females, which may expose males to more diverse *E. coli* strains. Indeed, similar effects have been described for other infectious agents [23]. In addition, differences in immune responses between males and females due to the hormonal challenges of male reproductive states may also underlie sex differences in *E. coli* infection [20], [21], [22]. That said, two prior studies of marsupials found no sex differences in *E. coli* infection patterns [55], [101] and it remains to be seen whether sex differences in *E. coli* infection are common across vertebrate species.

We also found effects of host age on *E. coli* populations. Specifically, elephants born around the same time were infected with genetically more similar *E. coli* than pairs of elephants further apart in age. This pattern occurred for pairs of elephants drawn from the same host population, as well as for pairs drawn from the two different populations (i.e., Amboseli and Samburu). One explanation for these effects is that they are caused by age-related changes in gut morphology or immune function leading to differences in the composition of phylogroups in younger versus older elephants. While this explanation is possible, it is not well supported by our data, as we found few changes in phylogroup composition as a function of age.

Instead, we think our results are more consistent with the idea that age-related patterns of *E. coli* infection reflect temporal structure in environmental *E. coli*. Specifically, individual hosts may be infected with *E. coli* when in the first few months of life, and while individuals are continually exposed to *E. coli* throughout life, most of these later strains may fail to establish [50]. In support, several studies have shown that temporal effects on genetic variation appear to be a dominant force in the population structure of *E. coli* within hosts and in the environment [55], [99], [102], [103]. For instance, when *E. coli* was sampled from several locations in a lake repeatedly for several days or years, there was strong genetic similarity among isolates collected on the same day or the same year across different locations separated by distances of 50 kilometers, as compared to *E. coli* collected on different days or years in the same location [102], [104]. Similar effects have been observed in *E. coli* populations infecting animal hosts [99], [101], [102], [103]. Our results appear to be novel with respect to the time scale as temporal effects on *E. coli* population structure have typically been explored over the scale of weeks or months, not decades. Given that elephant births are often seasonally clustered, and habitat and rainfall conditions can vary markedly between birth cohorts over their first years of life, these variations may affect transmission during the early acquisition of *E. coli*. That said, the time scale over which we observed temporal patterns on *E. coli* communities is surprising, given that B1 strains, which were the most common in our study, are among the most transient members of the gut. It is possible that B1 phylogroups behave differently in elephants as compared to other hosts. However, our results require further confirmation to see if they will be upheld. We encourage future researchers to test for similar temporal patterns within host populations.

### Conclusions

Social structure is often proposed to be an important conduit for the transmission of directly and environmentally transmitted infectious agents. However, our results indicate that social structure plays, at most, a weak role in *E. coli* transmission in wild elephants. Instead, transmission patterns were dominated by host habitat and aspects of individual hosts, such as individual sex and age. Strong patterns of socially mediated transmission may be limited to infectious agents with high host specificity, transmitted only via physical contact between hosts, and to hosts with social structures that minimize habitat overlap and contact between different group members. Generalist infectious agents that can be transmitted through the environment, such as *E. coli*, are common in wildlife, and they may be some of the most important from an evolutionary and a management perspective. In the future, we encourage researchers to incorporate multiple aspects of hosts and their environment, as well as social contacts, to gain the greatest insight into transmission patterns.

## Supporting Information

Figure S1 **Nucleotide diversity in** ***E. coli*** **as a function of host sex**. Boxplots depict nucleotide diversity in *E. coli* infecting individual elephants as a function of host sex. This analysis was performed on adult animals from Samburu NR only.
(TIF)

Figure S2 **The distribution of** ***E. coli*** **phylogroups groups as a function of host age.** Plot depicts a cross-sectional analysis of the proportion of each phylogroup type found infecting elephant hosts of different ages. Relationships demonstrate that unclassified *E. coli* isolates increased in older age groups.
(TIF)

Table S1 Sample information, including the number of elephant hosts per family group, the number of *E. coli* isolates genotyped per family group, and the number of GPS sightings per family group.
(DOCX)

Table S2 Percent of *E. coli* isolates assigned to different phylogroups in Amboseli and Samburu. Unassigned isolates did not cluster with any known ECOR sequences.
(DOCX)

Table S3 Basic evolutionary parameter estimates for concatenated *E. coli* sequences.
(DOCX)
