# Supplementary figures and images for: The Influence of Social Structure, Habitat, and Host Traits on the Transmission of Escherichia coli in Wild Elephants
Source: PLoS One. 2014 Apr 4;9(4):e93408. doi: 10.1371/journal.pone.0093408 (PMC3976290; doi:10.1371/journal.pone.0093408)

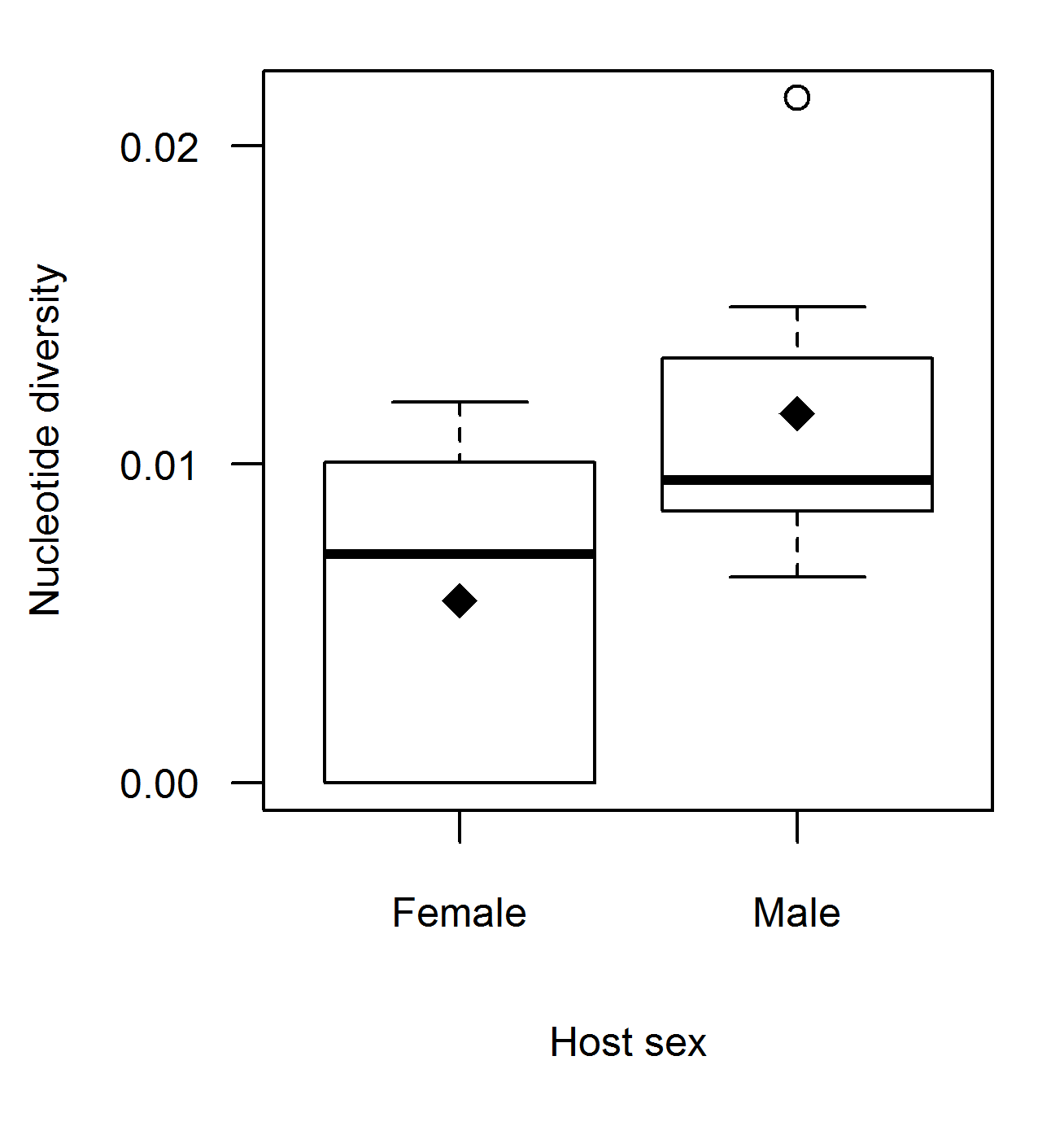

Supplement: Figure S1 — Nucleotide diversity in E. coli as a function of host sex. Boxplots depict nucleotide diversity in E. coli infecting individual elephants as a function of host sex. This analysis was performed on adult animals from Samburu NR only. (TIF) [file pone.0093408.s001.tif]

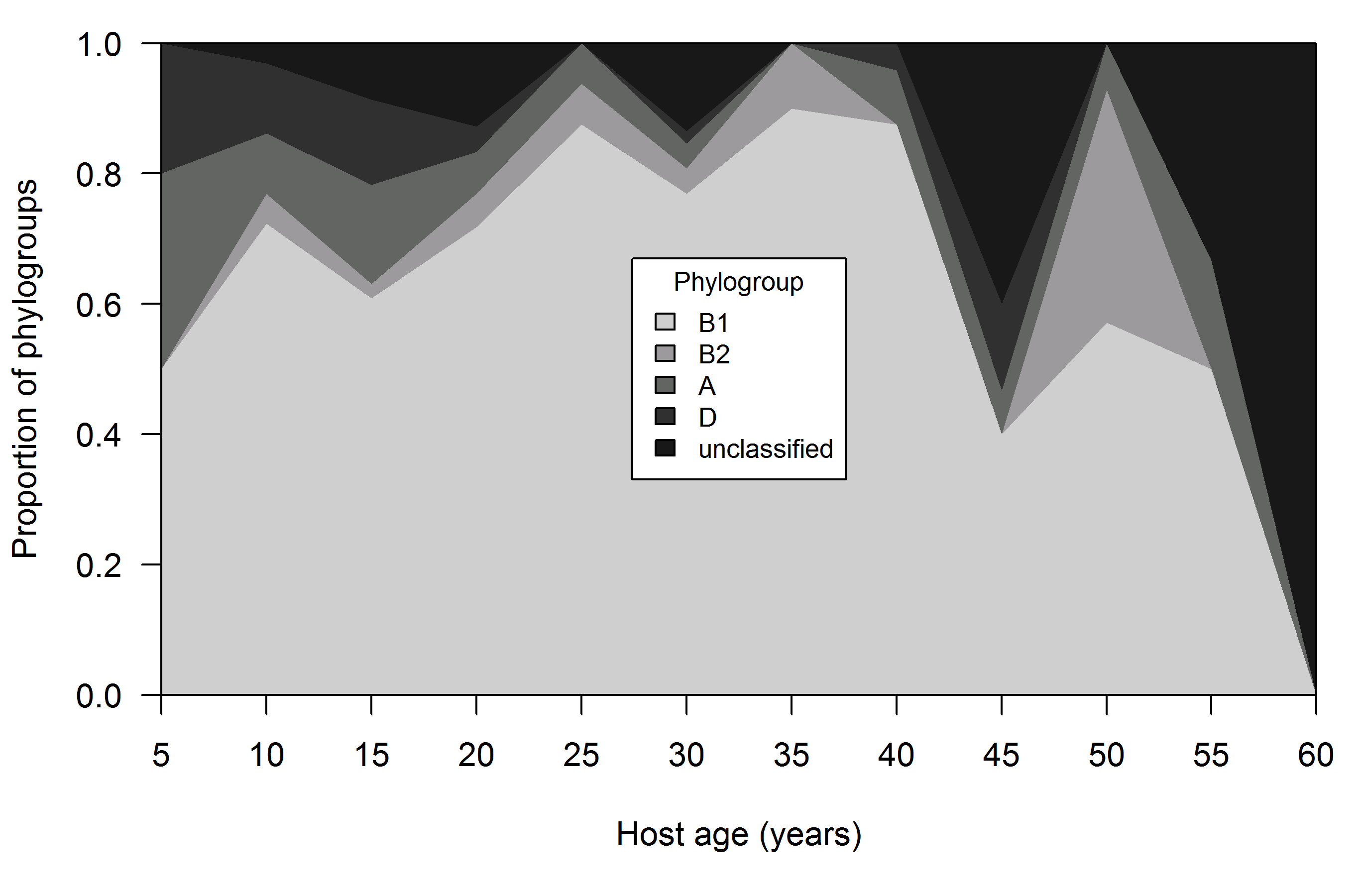

Supplement: Figure S2 — The distribution of E. coli phylogroups groups as a function of host age. Plot depicts a cross-sectional analysis of the proportion of each phylogroup type found infecting elephant hosts of different ages. Relationships demonstrate that unclassified E. coli isolates increased in older age groups. (TIF) [file pone.0093408.s002.tif]
