# Supplementary material for: The Influence of Social Structure, Habitat, and Host Traits on the Transmission of Escherichia coli in Wild Elephants
Source: PLoS One. 2014 Apr 4;9(4):e93408. doi: 10.1371/journal.pone.0093408 (PMC3976290; doi:10.1371/journal.pone.0093408)
Supplement: Table S1 — Sample information, including the number of elephant hosts per family group, the number of E. coli isolates genotyped per family group, and the number of GPS sightings per family group. (DOCX) [file pone.0093408.s003.docx]

| Family name | Number of family members | Number (and %) of individuals sampled for *E. coli* | Number of *E. coli* isolates genotyped | Number of GPS Sightings in a 5-year span |
| --- | --- | --- | --- | --- |
| *Amboseli* |  |  |  |  |
| AA | 21 | 12 (57.14) | 26 | 207 (196.65) |
| AC | 7 | 3 (42.86) | 6 | 50 (47.50) |
| BB | 24 | 11 (45.83) | 22 | 150 (142.50) |
| CB | 11 | 7 (63.64) | 13 | 203 (192.85) |
| EA | 25 | 10 (40.00) | 13 | 127 (120.65) |
| EB | 31 | 12 (38.71) | 32 | 226 (214.70) |
| FB | 19 | 6 (31.58) | 32 | 114 (108.30) |
| GB | 31 | 6 (19.35) | 12 | 220 (209) |
| IAC | 13 | 6 (46.15) | 29 | 62 (58.90) |
| OA | 25 | 12 (48.00) | 27 | 232 (220.40) |
| *Mean* | *20.7* | *8.5 (43.33)* | *21.2* | *159* |
|  |  |  |  |  |
| *Samburu* |  |  |  |  |
| Artists | 18 | 6 (33.33) | 25 | 124 (117.8) |
| Butterflies | 10 | 6 (60.00) | 23 | 88 (83.6) |
| Planets | 21 | 5 (23.81) | 20 | 40 (38) |
| Royals | 12 | 6 (50.00) | 24 | 137 (130.15) |
| Spices | 10 | 6 (60.00) | 24 | 81 (76.95) |
| *Mean* | *14.2* | *5.8 (45.43)* | *23.2* | *94* |
|  |  |  |  |  |
| Adult males | NA | 7 | 27 | - |
