# Supplementary material for: The Influence of Social Structure, Habitat, and Host Traits on the Transmission of Escherichia coli in Wild Elephants
Source: PLoS One. 2014 Apr 4;9(4):e93408. doi: 10.1371/journal.pone.0093408 (PMC3976290; doi:10.1371/journal.pone.0093408)
Supplement: Table S2 — Percent of E. coli isolates assigned to different phylogroups in Amboseli and Samburu. Unassigned isolates did not cluster with any known ECOR sequences. (DOCX) [file pone.0093408.s004.docx]

| **Phylogroup** | **Amboseli** | **Samburu** |
| --- | --- | --- |
| B1 | 72.9% | 64.3% |
| A | 9.5% | 9.1% |
| B2 | 1.9% | 9.1% |
| D | 7.6% | 5.6% |
| Unassigned | 8.1% | 11.9% |
