# Supplementary material for: The Influence of Social Structure, Habitat, and Host Traits on the Transmission of Escherichia coli in Wild Elephants
Source: PLoS One. 2014 Apr 4;9(4):e93408. doi: 10.1371/journal.pone.0093408 (PMC3976290; doi:10.1371/journal.pone.0093408)
Supplement: Table S3 — Basic evolutionary parameter estimates for concatenated E. coli sequences. (DOCX) [file pone.0093408.s005.docx]

| Variable | Amboseli | | Samburu | | Combined | |
| --- | --- | --- | --- | --- | --- | --- |
|  | Mean | 95% Credible intervals | Mean | 95% Credible intervals | Mean | 95% credible intervals |
| Mutation events (θ) | 27 | 11 – 45 | 41 | 23 – 63 | 53 | 34 – 74 |
| Recombination events | 57 | 38 – 80 | 41 | 29 – 57 | 86 | 66 – 108 |
| Mean tract Length (δ) | 232 | 157 – 328 | 201 | 133 – 294 | 196 | 147 – 257 |
| R/M | 4.478 | 2.451 – 8.833 | 2.650 | 1.450 – 4.414 | 3.331 | 2.063 – 5.251 |
| ρ/θ | 2.324 | 1.112 – 4.960 | 1.073 | 0.541 – 1.986 | 1.702 | 0.995 – 2.863 |
| TMRCA | 0.616 | 0.3650 –1.132 | 0.516 | 0.357 – 0.782 | 0.393 | 0.272 – 0.538 |
